# A novel, rationally designed, hybrid antimicrobial peptide, inspired by cathelicidin and aurein, exhibits membrane-active mechanisms against *Pseudomonas aeruginosa*

**DOI:** 10.1038/s41598-020-65688-5

**Published:** 2020-06-04

**Authors:** Natthaporn Klubthawee, Poom Adisakwattana, Warunee Hanpithakpong, Sangdao Somsri, Ratchaneewan Aunpad

**Affiliations:** 1 Graduate Program in Biomedical Sciences, Faculty of Allied Health Sciences, Thammasat University, Pathum Thani, Thailand; 2 Department of Helminthology, Faculty of Tropical Medicine, Mahidol University, Bangkok, Thailand; 3 Department of Clinical Pharmacology, Mahidol Oxford Tropical Medicine Research Unit, Faculty of Tropical Medicine, Mahidol University, Bangkok, Thailand

**Keywords:** Microbiology, Antimicrobials, Antibiotics

## Abstract

Antimicrobial peptides (AMPs) are promising alternatives to classical antibiotics for the treatment of drug-resistant infections. Due to their versatility and unlimited sequence space, AMPs can be rationally designed by modulating physicochemical determinants to favor desired biological parameters and turned into novel therapeutics. In this study, we utilized key structural and physicochemical parameters, in combination with rational engineering, to design novel short α-helical hybrid peptides inspired by the well-known natural peptides, cathelicidin and aurein. By comparing homologous sequences and abstracting the conserved residue type, sequence templates of cathelicidin (P0) and aurein (A0) were obtained. Two peptide derivatives, P7 and A3, were generated by amino acid substitution based on their residue composition and distribution. In order to enhance antimicrobial activity, a hybrid analog of P7A3 was designed. The results demonstrated that P7A3 had higher antibacterial activity than the parental peptides with unexpectedly high hemolytic activity. Strikingly, C-terminal truncation of hybrid peptides containing only the α-helical segment (PA-18) and shorter derivatives confer potent antimicrobial activity with reduced hemolytic activity in a length‐dependent manner. Among all, PA-13, showed remarkable broad-spectrum antibacterial activity, especially against *Pseudomonas aeruginosa* with no toxicity. PA-13 maintained antimicrobial activity in the presence of physiological salts and displayed rapid binding and penetration activity which resulted in membrane depolarization and permeabilization. Moreover, PA-13 showed an anti-inflammatory response via lipopolysaccharide (LPS) neutralization with dose-dependent, inhibiting, LPS-mediated Toll-like receptor activation. This study revealed the therapeutic potency of a novel hybrid peptide, and supports the use of rational design in development of new antibacterial agents.

## Introduction

The increasing emergence and dissemination of antibiotic resistance among bacterial pathogens has become a global public health challenge^1,2^. Therefore, there is an urgent need to develop new antimicrobial agents to overcome this problem. Antimicrobial peptides (AMPs) are an essential component of the innate immune system produced as a first line of defense by all multicellular organisms^3^. With such exceptional properties as broad-spectrum antimicrobial activity, rapid action and infrequent development of resistance^4,5^, AMP-based pharmaceuticals provide excellent templates for a wide range of antimicrobial agents and biomedical applications.

In general, naturally occurring AMPs are between 12 and 50 amino acids in length, and often contain cationic and hydrophobic residues^6^. Previous studies contributing to the understanding of the structure-activity relationship of AMPs show that one important class of membrane-active AMPs infers an amphipathic α-helical conformation^4,7,8^. The initial electrostatic interaction between a positively-charged AMP and the negatively-charged microbial cell membrane, such as lipoteichoic acids in Gram-positive bacteria or lipopolysaccharides (LPSs) in Gram-negative bacteria, results in insertion of a hydrophobic segment into the lipid bilayer of the microbial membranes. The effect is to destabilize by pore formation and produce cell death, a process unlike that of conventional antibiotics which have specific modes of action such as inhibition of cell wall or nucleic acid synthesis^9^.

Cathelicidins, a prominent family of AMPs, play important roles in the innate immune system of practically all species of vertebrates^10^. The α-helical cathelicidins possess broad‐spectrum antimicrobial activity against bacteria, fungi and viruses, and of note also against antibiotic-resistant strains of pathogenic bacteria^11^. Besides their antimicrobial activity, cathelicidins bind and neutralize LPS, and protect against endotoxic shock in a murine model of septicemia^12^. Frog skin is a natural storehouse of active AMPs^13^. Upon contact with microorganisms, anuran skin peptides are produced in dermal serous glands and stored within granules for release onto the skin surface after stress or tissue injury as part of the immune system^14^. Amongst them, aurein peptides from the Australian southern bell frogs *Litoria aurea* and *Litoria raniformis*^15^, are a large family of peptides with prominent activity toward both bacteria and cancer cells^16^.

Despite the many desirable properties of AMPs, they also exhibit undesirable properties including hemolytic activity toward human red blood cells, sensitivity to protease, salt and serum, and high production cost which impede their development as therapeutic agents^17^. However, optimization of sequences or modification of AMPs can improve antimicrobial effects while reducing cytotoxicity and so overcome barriers and expedite development for clinical application^7,18–20^. Due to their versatility and unlimited sequence space, AMPs can be rationally designed by modulating physicochemical determinants to favor the desired biological parameters and turned into novel therapeutics. Truncation, hybrid analog modification, and redesign by amino acid substitution of native peptide sequences provide a simple and effective approach for developing new antimicrobial agents. The antimicrobial activity of GI24, a 24-residue truncated peptide of PMAP-36, is not affected by the truncation of the C-terminal region^8^. The truncation of LL-37, based on the amino acid composition and 3D structure, retains its antimicrobial activity while losing its side effects^21^. The synthetic hybrid peptides of progetrin-1, bovine lactoferricin and cecropin A, LB-PG and CA-PG, exhibit broad antimicrobial activity against both Gram-positive and -negative bacteria along with reduced hemolytic activity^19^. An embedded-hybrid peptide R-FV-I16 has potent antimicrobial and anti-biofilm activity, with less hemolytic activity and cytotoxicity^22^. Modification of the hybrid peptide of cecropin A and LL-37 improves the therapeutic index^23^.

In the present study, we utilized key structural and physicochemical parameters in combination with rational engineering to design novel short, α-helical hybrid peptides inspired by the well-known natural α-helical peptides, cathelicidin and aurein. By multiple sequence alignment of 45 α-helical cathelicidins and 11 α-helical aureins, and a template-modified strategy, two novel peptides (P0 and A0) were designed from the conserved sequences. They were used as scaffolds to design a series of derivatives by amino acid substitution, hybrid analog modification and truncation. The novel synthetic α-helical hybrid peptide, PA-13, showed antimicrobial activity against a broad range of microbes including multidrug-resistant (MDR) *Pseudomonas aeruginosa* clinical isolates with no toxicity toward human red blood cells and L929 cells. The mechanism of action at the membrane level and anti-inflammatory activity of PA-13 were determined. Our current study highlighted the potential for rational design of short α-helical peptides which provide a first step towards development of novel antimicrobial agents.

## Materials and methods

### Peptide design and sequence analysis

Inspired by natural α-helical AMPs, a template-modified strategy was exploited to design short α-helical AMPs^20^. Sequence and structural information from α-helical cathelicidin and aurein were used to build and design a series of derivatives with improved antimicrobial activities and lessened toxicities. P0 and A0 parent peptides were designed from the conserved sequences of 45 α-helical cathelicidins (Fig. S1; Table S1) and 11 α-helical aureins (Fig. S2; Table S2), respectively, retrieved from the APD (http://aps.unmc.edu/AP/main.php). Based on its α-helical secondary structure and helical wheel projection (Fig. S3), P7 peptide was designed from the sequence of P0 peptide by truncating the unstructured region from position 1 to position 3, then substituting in three hydrophobic amino acids at positions 6, 10 and 13 (K6A, G10W and D13L), and one positively charged amino acid (L13R). A3 peptide was modified from A0 peptide by substituting three positions with arginine (G1R, D4R and I5R). Fragments of P7 and A3 were *in silico* combined to obtain a novel hybrid peptide, P7A3. Then a series of derivatives, containing only the α-helical segment (PA-18) and shorter peptides (PA-17, PA-16, PA-15, PA-14 and PA-13), were designed by truncating amino acids at the C-terminal of P7A3. All these peptide derivatives (Table S3) underwent post-translational modification by C-terminal amidation.

The peptide sequences were analyzed primarily using the programs ProtParam (ExPASy Proteomics Server: http://www.expasy.org/tools/protparam. html) and Antimicrobial Peptide Calculator and Predictor (APD3 Server: http://aps.unmc.edu/AP/prediction/ prediction_main.php). A three-dimensional structure was predicted by I-TASSER (http://zhanglab.ccmb.med.umich.edu/I-TASSER/). The helical wheel projection was calculated by using the online program NetWheels: Peptides Helical Wheel and Net projections maker (http://lbqp.unb.br/NetWheels/)^8^.

### Peptide synthesis

All of the peptides including TAMRA-labelled PA-13 were synthesized by solid-phase methods using 9-fluorenylmethoxycarbonyl (Fmoc) chemistry and purified by HPLC as trifluoroacetate salts (ChinaPeptides, China). The content of residual TFA, quantified by ^19^F nuclear magnetic resonance (NMR), was less than 1.7% (wt/wt). The TAMRA-labelled PA-13 was prepared through the method of dehydration condensation and TAMRA was linked with PA-13 via an amide bond at the N-terminus. The purity of all the peptides was more than 98% as ascertained by analytical reversed-phase HPLC. Electrospray ionization mass spectrometry (ESI-MS) was used to identify the peptides.

### Antimicrobial assay

The antimicrobial activity of the peptides was determined against both Gram-positive and -negative bacteria including 14 MDR *P. aeruginosa* clinical isolates from Bhumibol Adulyadej Hospital (Tables 1 and 3). The minimal inhibitory concentration (MIC) of each peptide was measured according to a modification of the National Committee for Clinical Laboratory Standards (NCCLS) broth microdilution method as previously described^24^. Briefly, bacterial cells in mid-log phase were cultured in Müeller-Hinton broth (MHB) and then diluted to 10^7^ CFU/ml. Fifty microliters of 2-fold serially diluted peptides, with concentrations ranging from 0.98 to 250 μg/ml in 0.01% (vol/vol) acetic acid and 0.2% (wt/vol) bovine serum albumin (BSA, Sigma), were added to each well of sterile 96-well plates, followed by 50 μl of bacterial solution. Plates were incubated at 37 °C for 24 h, and MICs were defined as the lowest concentration of peptide that prevented visible turbidity. Cultures without peptides and uninoculated MHB were employed as positive and negative controls, respectively. Colony count assays were performed to determine the minimal bactericidal concentrations (MBCs) for determination of time-killing activity. As previously described^25^, a 50 μl aliquot from each non-turbid well identified in the MIC determination experiment was spread on agar plates. MBCs were defined as the lowest concentration of peptides where no colony growth was observed on agar plates after 24 h incubation at 37 °C.

**Table 1:** Minimum inhibitory concentration (MIC) of PA-13 compared with ciprofloxacin, gentamycin, imipenem and colistin against 14 strains of multidrug-resistant (MDR) *Pseudomonas aeruginosa* clinical isolates.

Bacterial strains	MIC (µg/ml)				
	PA-13	Ciprofloxacin	Gentamycin	Imipenem	Colistin
**Standard strain**					
*P. aeruginosa* ATCC 27853	3.91	≤0.98	≤0.98	≤0.98	≤0.98
**Clinically isolated MDR** ***P. aeruginosa***					
*P. aeruginosa* No.1	3.91	62.5	>125	>125	1.95
*P. aeruginosa* No.2	7.81	250	>125	>125	1.95
*P. aeruginosa* No.3	7.81	250	>125	>125	1.95
*P. aeruginosa* No.4	7.81	250	>125	>125	1.95
*P. aeruginosa* No.5	7.81	62.5	125	125	1.95
*P. aeruginosa* No.6	7.81	250	>125	>125	1.95
*P. aeruginosa* No.7	7.81	125	125	>125	0.98
*P. aeruginosa* No.8	7.81	125	>125	125	0.98
*P. aeruginosa* No.9	7.81	250	>125	>125	0.98
*P. aeruginosa* No.10	7.81	31.25	>125	>125	1.95
*P. aeruginosa* No.11	7.81	31.25	>125	>125	1.95
*P. aeruginosa* No.12	3.91	31.25	125	125	1.95
*P. aeruginosa* No.13	15.63	62.5	>125	62.5	0.98
*P. aeruginosa* No.14	7.81	31.25	>125	>125	0.98

**Table 2:** MIC values of PA-13 in the presence of physiological salts against *P. aeruginosa* ATCC 27853.

Peptides	Physiological salts^a^							
	Control^b^	NaCl	KCl	MgCl_2_	NH_4_Cl	ZnCl_2_	FeCl_3_	CaCl_2_
PA-13	3.91	31.25	7.81	31.25	3.91	3.91	3.91	3.91

^a^The final concentrations of NaCl, KCl, MgCl_2_, NH_4_Cl, ZnCl_2_, FeCl_3_, and CaCl_2_ were 150 mM, 4.5 mM, 1 mM, 6 µM, 8 µM, 4 µM and 2.5 µM, respectively.

^b^Control MIC values were determined in the absence of these physiological salts.

### Measurement of hemolytic activity

Hemolytic activity of the peptides was assayed against human red blood cells (hRBCs) by measuring the amount of hemoglobin released after treatment^26^. The hRBCs, freshly collected from a healthy volunteer in polycarbonate tubes containing heparin, were washed three times in sterile phosphate buffered saline (PBS) and centrifuged at 2,000 × *g* for 5 min or until the supernatant became clear. The hRBCs were diluted to a final concentration of 2% (vol/vol), then 50 µl of the hRBCs suspension was incubated with 50 µl of different concentrations (0.98 to 250 μg/ml) of a peptide dissolved in PBS. After 1 h of incubation at 37 °C, intact hRBCs were pelleted by centrifugation at 2,000 × *g* for 10 min. The supernatant was transferred to a new 96-well plate and the release of hemoglobin was monitored by measurement of absorbance at 405 nm using a Multiskan FC microplate reader. The hRBCs in PBS only (OD_Blank_) and in 0.1% Triton X-100 (OD_Triton X-100_) were employed as negative (0% hemolysis) and positive (100% hemolysis) controls, respectively. The percentage of hemolysis was calculated according to the following equation:

$$ \% \,{\rm{Hemolysis}}=({{\rm{OD}}}_{{\rm{Sample}}}-{{\rm{A}}}_{{\rm{Blank}}})/({{\rm{OD}}}_{{\rm{TritonX}}-100}-{{\rm{OD}}}_{{\rm{Blank}}})\times 100$$

### Ethics statement

The experimental protocol involving human participants was carried out in accordance with the ethical standards and approved by the Ethics Committee of Thammasat University (COA No. 066/2562). Informed consent was obtained from all individual participants involved in the present study.

### Cytotoxicity assay

The cytotoxicity of PA-13 on L929 mouse fibroblast cells was assayed using the colorimetric 3-(4,5 dimethylthiazol-2-yl)-2,5-diphenyltetrazolium bromide (MTT) (Sigma) dye reduction assay according to a previously described method^27^. L929 cells were cultured in RPMI 1640 supplemented with 10% (vol/vol) FBS, 0.2% (wt/vol) sodium bicarbonate, 2 mM L-glutamine, 100 U/ml penicillin and 100 mg/ml streptomycin, then the cells were maintained in a humidified incubator with 5% CO_2_ at 37 °C. After L929 cells were seeded into 96-well plates at a density of 3 × 10^4^ cells per well, a peptide was added at different concentrations (0.98 to 250 μg/ml) and plates were incubated at 37 °C under 5% CO_2_ for 24 h. Medium with and without cells was used as positive and negative controls, respectively. Following incubation, 10 μl of MTT (5 mg/ml) was added to each well and incubated at 37 °C under 5% CO_2_ for 4 h. After the metabolically active cells converted the yellow MTT to purple formazan, the supernatants of cell cultures were discarded, then 150 μl of dimethyl sulfoxide (DMSO) was added to each well and mixed gently to dissolve the formazan crystals. The absorbance was monitored at 570 nm using a Tecan microplate reader. The percentage of viable cells was calculated according to the following equation:

$$ \% \,{\rm{Viability}}=({{\rm{OD}}}_{{\rm{Treated}}}-{{\rm{OD}}}_{{\rm{Blank}}})/({{\rm{OD}}}_{{\rm{Untreated}}}-{{\rm{OD}}}_{{\rm{Blank}}})\times 100$$

### Time-killing analysis

The bacterial killing kinetics of PA-13 was assessed by evaluating the time course to kill suspensions of *P. aeruginosa* ATCC 27853 as described previously^28^. Bacteria in mid-logarithmic phase (10^7^ CFU/ml) were incubated with PA-13 at certain concentrations (MIC and MBC) in MHB at 37 °C. 10 μl of bacterial suspensions were removed at various time intervals (0.5, 1, 2, 3, 4, 5, 6 and 7 h), ten-fold serially diluted in MHB, and 100 µl of each dilution was plated onto tryptic soy agar (TSA). Plates were incubated at 37 °C to determine the number of CFU after 24 h; the assays were performed in triplicate.

### Circular dichroism (CD) analysis

The secondary structure of PA-13 in different environments was determined on a Jasco-815 spectropolarimeter (Jasco, Tokyo, Japan) at 25 °C, using a 0.1-cm-path-length rectangular quartz cell as described previously^19^. To investigate conformational changes of PA-13 induced by membrane mimic environments, the peptide was recorded at a final concentration of 0.2 mg/ml in a mixture of deionized (DI) water (mimicking an aqueous environment), 30 mM SDS micelles [mimicking negatively charged prokaryotic membrane (Sigma)], 50% (vol/vol) TFE (2,2,2-trifluoroethanol) [giving an environment comparable to the hydrophobic compartment of microbial membranes (Sigma)] and 0.05% (wt/vol) lipopolysaccharide (LPS) from *P. aeruginosa* serotype 10 (Sigma). The spectra were recorded over wavelengths of 190 and 250 nm at a scanning speed of 10 nm/min. At least three scans were conducted for each condition. The acquired circular dichroism (CD) signal spectra were then converted to mean residue ellipticity with the following equation:

$${{\rm{\theta }}}_{{\rm{M}}}=({{\rm{\theta }}}_{{\rm{obs}}}/10)\times ({{\rm{M}}}_{{\rm{RW}}}/{\rm{c}}\cdot 1)$$

where θ_M_ is residue ellipticity (deg. M^−1^ m^−1^), θ_obs_ is the observed ellipticity corrected for the buffer at a given wavelength (mdeg), M_RW_ is residue molecular weight (M_W_/number of amino acids), c is peptide concentration (mg/ml), and l is the path length (cm).

### Salt sensitivity

To test the effect of salts on the antibacterial activity of PA-13, the mid-logarithmic phase (10^7^ CFU/ml) of *P. aeruginosa* ATCC 27853 was exposed to different concentrations of PA-13 in the presence of different physiological salts: 150 mM NaCl; 4.5 mM KCl; 1 mM MgCl_2_; 6 µM NH_4_Cl; 8 µM ZnCl_2_; 4 µM FeCl_3_; 2.5 µM CaCl_2_^22^. After these treatments, the MICs were determined as described above.

### Flow cytometry

#### Membrane permeability and depolarization

Their outer and inner membranes provide important protection for Gram-negative bacteria. To assess membrane permeability and depolarization induced by PA-13, the DNA intercalating dyes propidium iodide (PI) and bis-(1,3-dibutylbarbituric acid) trimethine oxonol (BOX) were used as previously described^29^. The fluorescence conferred by these dyes is generally associated with the loss of membrane integrity and potential, respectively.

Briefly, *P. aeruginosa* ATCC 27853 was cultured to mid-logarithmic phase and harvested by centrifugation at 2,000 × g for 5 min. The bacterial cells were washed thrice with PBS and diluted to 10^7^ CFU/ml in 50 ml of PBS. The bacterial suspension was treated with PA-13 at concentrations of 0.5 × MIC and 1 × MIC, then incubated for 15, 30 or 60 min at 37 °C with constant shaking at 140 rpm. Bacterial pellets were harvested and washed with PBS by centrifugation at 10,000 × g for 10 min. PI (final concentration of 1 µg/ml, Sigma) and BOX (final concentration of 0.0625 µM, Sigma) were added to each sample, with unbound dye removed by washing with PBS^8^. Untreated bacterial cells (no peptide) served as a negative control, whereas bacterial cells treated with melittin served as a positive control. Data was recorded using a flow cytometer (CytoFlex, Beckman Coulter) at a laser excitation wavelength of 488 nm. Forward scatter (FS), side scatter (SS), green fluorescence (530/30 nm) from BOX and red fluorescence (585/42 nm) emitted by PI were collected using logarithmic scales. 25,000 cells, defined according to their scatter parameters, were counted in each sample. The experiments were performed in triplicate and the data was analyzed using Kaluza software.

#### Membrane-penetrating activity

To clarify whether bacterial membrane permeabilization resulted from peptide uptake, the interaction of TAMRA-labelled PA-13 with the membrane of bacteria cells was investigated by flow cytometry^27^. Mid-logarithmic phase suspensions of *P. aeruginosa* ATCC 27853 were incubated with TAMRA-labelled PA-13 at 1 × MIC, and incubated for 5, 10, 15, 30 or 60 min at 37 °C. After incubation, unbound labelled peptide was removed by washing with PBS. Then the fluorescence signal from treated cells was determined by flow cytometry (CytoFlex, Beckman Coulter).

### Localization of TAMRA-Labelled PA-13

To investigate how PA-13 is localized within the bacterial cell, confocal microscopy was used to study *P. aeruginosa* ATCC 27853 after incubation with TAMRA-labelled PA-13. Briefly, mid-logarithmic phase *P. aeruginosa* were harvested by centrifugation at 2,000 × g for 5 min, washed twice in PBS, and resuspended in the same buffer. Approximately 1 × 10^8^ cells were incubated with TAMRA-labelled PA-13 at 1 × MIC for 30 min at 37 °C. After incubation, unbound labelled peptide was removed by washing with PBS. The samples were fixed with 4% paraformaldehyde for 20 min, washed twice in PBS, and mixed with antifade (Prolong antifade reagent with DAPI, Invitrogen). Finally, the samples were mixed with 2% low melting agarose (Sigma) on glass slides. Then, localization of PA-13 in the bacterial cells was observed under a confocal laser scanning microscopy (LSM 800 with Airyscan, ZEISS).

### Scanning electron microscopy (SEM)

The morphological changes of bacteria after treatment with PA-13 were observed under scanning electron microscopy (SEM). As previously described^8^, *P. aeruginosa* ATCC 27853 was cultured in tryptic soy broth (TSB) to mid-logarithmic phase, and harvested by centrifugation at 2,000 × g for 5 min. Cells pellets were washed twice with PBS and resuspended to an OD_620_ of 0.2. The cell suspension was incubated at 37 °C for 120 min with PA-13 at 0.5 × MIC. After incubation, the cells were centrifuged at 10,000×g for 20 min and washed twice with PBS. Bacterial pellets were then fixed in 2.5% (vol/vol) glutaraldehyde at 4 °C overnight. Then the samples were washed twice in PBS and dehydrated in a graded ethanol series (50%, 70%, 90%, and 100%), each dilution for 15 min. Finally, all the samples were transferred to mixtures (1:1) of ethanol and tertiary butanol, and then to pure tertiary butanol, for 20 min each. The specimens were observed after lyophilization and gold coating using a scanning electron microscope (Hitachi SU8020, Japan).

### Transmission electron microscopy (TEM)

The bacterial cells were treated and prepared with the same conditions as previously described in SEM^8^. The bacterial pellets were washed twice with PBS and post-fixed with 1% osmium tetroxide in PBS for 2 h, after overnight fixing with 2.5% glutaraldehyde. The fixed bacterial cells were washed twice with PBS, followed by dehydration for 15 min in a graded ethanol series (50%, 70%, 90%, and 100%). After placing in absolute acetone for 20 min, these samples were transferred to a mixture of absolute acetone and epoxy resin (1:1 and 1:3) for 1 h each, followed by transfer to pure epoxy resin and held overnight. Ultrathin sections were cut with a glass knife on an ultramicrotome, and post-stained with uranyl acetate and lead citrate. Specimens were observed using a transmission electron microscope (Hitachi HT7700, Japan).

### LPS neutralization studies

#### LPS binding activity

The ability of PA-13 to bind LPS was assessed using a quantitative, chromogenic limulus amebocyte lysate (LAL) according to the manufacturer’s instructions (Thermo Scientific). The LAL test is a sensitive indicator of the presence of free or non-neutralized endotoxin^30^. Stock solutions of peptide were prepared in endotoxin-free water provided with the kit. Briefly, 1 EU/mL of LPS from *E. coli* O111:B4 was incubated with various concentrations of PA-13 (1 × MIC, 2 × MIC, 4 × MIC, 16 × MIC or 32 × MIC) at 37 °C for 30 min to allow binding. LAL reagent was added and incubated for 10 min at 37 °C, followed by addition of a colorless substrate and incubation for a further 6 min. The reaction was stopped with 25% (vol/vol) glacial acetic acid and the release of p-nitroaniline followed by absorbance at 405 nm. Endotoxin-free water served as a negative control (blank) and was considered 0% LPS binding. After blank subtraction, the percentage of LPS binding was calculated according to the following equation:

$${\rm{ \% }}\,{\rm{L}}{\rm{P}}{\rm{S}}\,{\rm{b}}{\rm{i}}{\rm{n}}{\rm{d}}{\rm{i}}{\rm{n}}{\rm{g}}=({{\rm{O}}{\rm{D}}}_{{\rm{U}}{\rm{n}}{\rm{t}}{\rm{r}}{\rm{e}}{\rm{a}}{\rm{t}}{\rm{e}}{\rm{d}}}{\text{-}{\rm{O}}{\rm{D}}}_{{\rm{T}}{\rm{r}}{\rm{e}}{\rm{a}}{\rm{t}}{\rm{e}}{\rm{d}}})/{{\rm{O}}{\rm{D}}}_{{\rm{U}}{\rm{n}}{\rm{t}}{\rm{r}}{\rm{e}}{\rm{a}}{\rm{t}}{\rm{e}}{\rm{d}}}\times 100$$

#### Effect of PA-13 on LPS-mediated TLR4 activation

The stimulation of human TLR4 (hTLR4) by activation of NF-kB was observed in HEK-Blue hTLR4 cells obtained by co-transfection of the hTLR4 gene, the MD-2/CD14 co-receptor genes and a secreted embryonic alkaline phosphatase (SEAP) reporter gene into HEK293 cells. Stimulation with LPS activates NF-kB and AP-1, inducing the production of SEAP. Opposing this, the peptide can interact with LPS and inhibit TLR4 signal transduction, and so decrease production of SEAP. The hydrolysis of substrate by SEAP produces a purple/blue color.

Briefly, HEK-Blue hTLR4 cells (3 × 10^4^ cells/well) were seeded in 96-well plates and incubated at 37 °C in an air atmosphere containing 5% CO_2_ for 24 h. These cells were further incubated with 250 ng/ml LPS of *P. aeruginosa* serotype 10 (Sigma) and different concentrations of PA-13 (0.98-250 µg/ml) for 16 h. After incubation, supernatants were collected and incubated with QUANTI-Blue™ (InvivoGen). The absorbance of SEAP was monitored at 620 nm. The results were calculated from at least three independent experiments.

## Results

### Peptide design and sequence analysis

**Figure 1:**
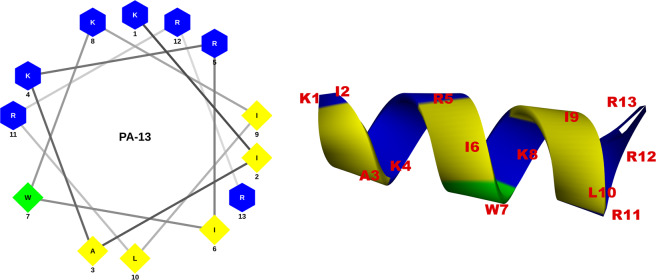
Helical wheel projections and three-dimensional structure simulation of PA-13 depicted in ribbon diagrams. Positively charged residues are presented in blue, hydrophobic residues are shown in yellow below the wheel and aromatic residues are shown in green. The numbers represented the position of amino acid residues.

Two parent α-helical peptides, P0 and A0, were designed from the conserved sequences of cathelicidin and aurein by a template-modified strategy. The structural and physicochemical parameters reported as key in designing novel antimicrobial peptides are hydrophobicity, amphipathicity and net charge^7^. Based on these parameters, P7 peptide was designed from the sequence of P0 peptide by truncating and substituting with three hydrophobic amino acids and one positively charged amino acid. A3 peptide was modified from A0 peptide by substituting with three arginines. P7 presented a perfect amphipathic structure with highest mean hydrophobic moment (Fig. S3; Table S3), whereas A3 showed an increase in positive charge from +1 to +6. A novel hybrid peptide, P7A3, was designed by *in silico* combining the P7 and A3 peptides. P7A3 displayed the highest positive charge with a cationic polar face and was unusually wide (Fig. S3). To identify the shortest amino acid sequence needed for potent antimicrobial activity and reduced cytotoxicity, a series of truncated derivatives were designed by truncating amino acids at the C-terminal end of P7A3. The 18-residue peptide, PA-18, contained the entire α-helical region of P7A3, while the shortest derivative (PA-13) also showed a helical structure in 3D structure projection (Fig. 1). The molecular weights of the peptides were verified by ESI-MS. All peptides had measured molecular weights consistent with their theoretical values, suggesting that the peptides were successfully synthesized (Table S3).

### Antibacterial and hemolytic activity

The MICs of all peptide derivatives against a wide range of Gram-negative and -positive bacterial strains were determined (Table 3). Peptides P0, A0, and A3 had no antibacterial activity against tested microorganisms, while P7 displayed potent antibacterial activity. Strikingly, the hybrid analogue (P7A3) showed higher antimicrobial activities than those of P7, especially against Gram-positive bacteria. The MICs of truncated derivatives of the hybrid analogue (PA-13, PA-14, PA-15, PA-16, PA-17 and PA-18) against both Gram-negative and -positive bacterial strains ranged from 1.95-250 μg/ml. PA-15 and PA-16 were the most active peptides against the Gram-negative and -positive bacterial strains tested, respectively. Among the hybrid peptides, the shortest peptide (PA-13) exhibited broad and potent antimicrobial activity against both Gram-positive and -negative bacteria, notably against *P. aeruginosa* and *Salmonella* Typhimurium ATCC 13311 compared with melittin.

**Table 3:** Minimum inhibitory concentration (MIC) of α-helical peptide derivatives against 12 strains of human pathogenic Gram-positive and -negative bacteria.

MIC (µg/ml)												
	P0	P7	A0	A3	P7A3	PA-18	PA-17	PA-16	PA-15	PA-14	PA-13	Melittin
**Gram-negative bacteria**												
*Pseudomonas aeruginosa* ATCC 27853	>250	7.81	>250	>250	7.81	7.81	7.81	7.81	7.81	3.91	3.91	15.63
*Escherichia coli* ATCC 25922	>250	15.63	>250	>250	15.63	31.25	15.63	7.81	15.63	15.63	31.25	31.25
*Escherichia coli* O157:H7 MT strain	>250	125	>250	>250	250	250	62.5	62.5	15.63	62.5	31.25	7.81
*Shigella sonnei* ATCC 11060	>250	3.91	>250	>250	3.91	3.91	3.91	3.91	3.91	3.91	3.91	3.91
*Salmonella* Typhimurium ATCC 13311	>250	7.81	>250	>250	7.81	3.91	3.91	1.95	1.95	1.95	1.95	3.91
*Vibrio cholera* O1 Inaba DMST 16261	>250	31.25	>250	>250	7.81	7.81	7.81	3.91	3.91	7.81	31.25	3.91
*Acinetobacter baumannii* MT strain	>250	7.81	>250	>250	7.81	15.63	7.81	7.81	3.91	3.91	7.81	7.81
**Gram-positive bacteria**												
*Staphylococcus aureus* ATCC 25923	>250	62.5	>250	>250	15.63	15.63	15.63	7.81	7.81	15.63	62.5	3.91
*Staphylococcus epidermidis* ATCC 12228	>250	3.91	>250	>250	3.91	3.91	3.91	3.91	3.91	3.91	3.91	3.91
*Bacillus cereus* ATCC 11778	>250	31.25	>250	>250	7.81	7.81	7.81	3.91	3.91	7.81	15.63	3.91
*Enterococcus faecalis* ATCC 29212	>250	>250	>250	>250	31.25	31.25	31.25	15.63	31.25	125	250	1.95
*Listeria monocytogenes* 10403 s	>250	7.81	>250	250	3.91	1.95	1.95	3.91	1.95	1.95	3.91	3.91
MHC^a^ (µg/ml)	>250	250	>250	>250	3.91	3.91	3.91	3.91	3.91	15.63	125	˂0.98
GM^b^ (Gr.− strains)	>250	28.46	>250	>250	42.97	45.76	15.63	13.67	7.54	14.23	15.90	10.60
Therapeutic index (TI)^c^ (Gr.− strains)	1.00	8.78	1.00	1.00	0.09	0.09	0.25	0.29	0.52	1.10	7.86	0.05
GM^d^ (Gr.+ strains)	>250	121.09	>250	>250	12.50	12.11	12.11	7.03	9.77	30.86	67.19	3.52
Therapeutic index (TI)^e^ (Gr.+ strains)	1.00	2.06	1.00	1.11	0.31	0.32	0.32	0.56	0.40	0.51	1.86	0.14

^a^MHC is the minimum concentration that caused 10% hemolysis of human red blood cells (hRBC). When no 10% hemolysis was observed at 250 µg/ml, a value of 500 µg/ml was used to calculate the therapeutic index.

^b^GM (Gr.- strains) denotes the geometric mean of MIC values from all Gram-negative strains.

^c^Therapeutic index (Gr.- strains) is the ratio of the MHC to the geometric mean of MICs from Gram-negative strains.

^d^GM (Gr.+ strains) denotes the geometric mean of MIC values from all Gram-positive strains.

^e^Therapeutic index (Gr.+ strains) is the ratio of the MHC to the geometric mean of MICs from all Gram-negative strains.

**Figure 2:**
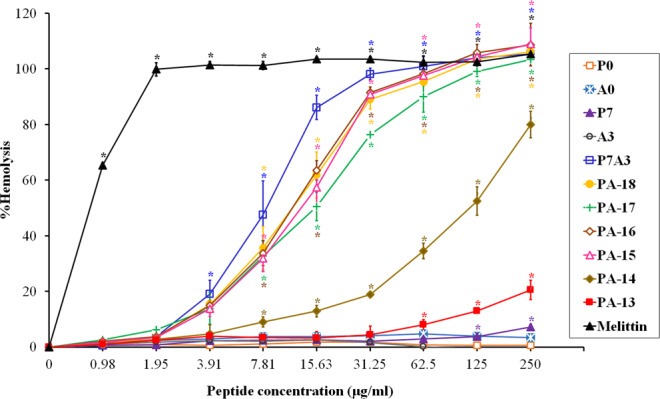
Hemolytic activity of peptide derivatives against human red blood cells. The experiments were performed in triplicate and the data were expressed as the mean ± SD. The statistical analysis was performed by one-way ANOVA and Tukey’s test at p < 0.05 level.

The hemolytic activity of peptides was tested by measuring their ability to lyse human RBCs at various concentrations (0.98-250 µg/ml) (Fig. 2). Melittin, which was used as a positive control, caused complete hemolysis at a concentration of 1.95 µg/ml. The parent peptides, P0 and A0, showed no obvious hemolytic activity even at maximum concentrations (less than 5% hemolysis). The hybrid peptide, P7A3, displayed stronger hemolytic activity than P7 and A3 alone, and its hemolysis reached 100% at a concentration of 62.5 µg/ml. After truncation, peptide PA-18 had decreased hemolytic activity, suggesting that the 5-amino-acid segment from position 19 to position 23 at the C-terminus was vital in maintaining the hemolytic activity of P7A3. The PA-18, PA-17, PA-16, PA-15 and PA-14 showed hemolytic activity in a dose-dependent manner. Among all truncated derivatives, PA-13 showed the least hemolytic activity. At the MIC, the hemolytic activity of PA-13 was less than 2%.

To further evaluate the global activity of these peptides, the geometric mean (GM) MIC and the therapeutic index (TI) of each were calculated (Table 3). The TI, a widely used parameter to evaluate cell selectivity and specificity of antimicrobial agents, was calculated as the ratio of MHC (the concentration that causes 10% hemolysis) to the geometric mean of MICs^22^. Higher TIs indicated greater cell selectivity and specificity. The TI values of PA-13 were comparable to those of P7, and about 7 to 90 times higher than those of all other peptide derivatives (Table 3). Taken together with the results of antibacterial and hemolytic activities, PA-13 was selected for further study because it had potent antimicrobial activity (notably against *Pseudomonas aeruginosa*) and a favorable safety profile.

The opportunistic pathogen *P. aeruginosa* is known to play a role in many types of infections, including lung infections, wound infections, otitis media and medical device-associated infections^31^. Treatment of *P. aeruginosa* infections is particularly challenged by intrinsic and acquired resistance to many conventional antibiotics^32^. The peptide PA-13 was active against all tested MDR *P. aeruginosa* isolates with MICs ranging from 3.91 to 15.63 μg/ml (Table 1). This was about 8-64 times higher than the antimicrobial activity of ciprofloxacin (31.25-250 μg/ml) which is an antibiotic commonly used for the treatment of *P. aeruginosa*. PA-13 was active against both drug-susceptible and MDR strains. We concluded that PA-13 has potent antibacterial activity against *P. aeruginosa*.

### Cytotoxicity of synthetic peptide

**Figure 3:**
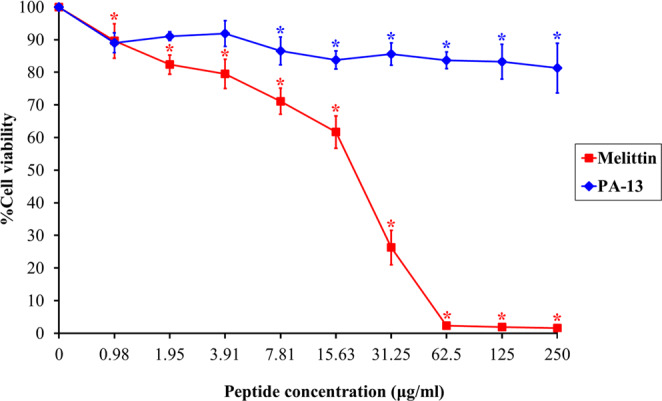
Cytotoxicity of PA-13 and melittin (positive control) against L929 cells. The experiments were performed in triplicate and the data were expressed as the mean ± SD. The statistical analysis was performed by one-way ANOVA and Tukey’s test at p < 0.05 level.

Cytotoxic activity of PA-13 against the L929 mouse fibroblast cell line was determined by a colorimetric MTT viability assay. Results are shown in Fig. 3. Melittin, used as a positive control, showed a strong and dose-dependent cytotoxic effect; no living cells were detected at 62.5 µg/ml. Peptide PA-13 displayed very little cytotoxicity on L929 cells when compared to melittin. At the MIC (3.91 µg/ml), the cell survival was up to 90%. Even at the maximum concentration (250 µg/ml), the survival was common (81.29%). On the contrary, melittin was very cytotoxic against L929 cells and dramatically reduced cell viability (less than 1%).

### Time-killing activity

**Figure 4:**
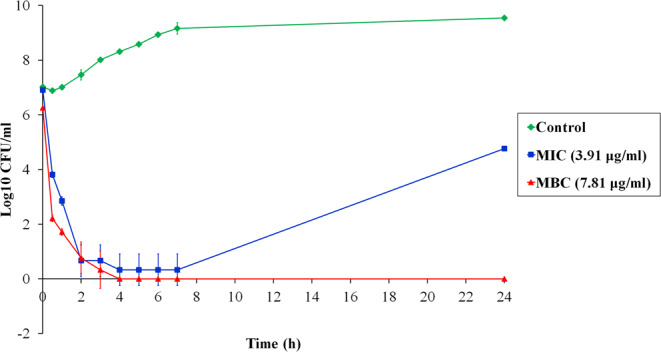
Time-killing kinetics of PA-13 against *P. aeruginosa* ATCC 27853 at MIC and MBC concentrations for 0, 0.5, 1, 2, 3, 4, 5, 6, 7 and 24 h.

A time-killing kinetic assay was used to evaluate the time course to kill bacteria in suspension. PA-13 displayed rapid killing with concentration- and time-dependent bactericidal activity (Fig. 4). Within 30 min, PA-13 reduced 10^7^ CFU/ml of *P. aeruginosa* to approximately 10^4^ CFU/ml and 10^2^ CFU/ml at MIC and MBC, respectively. In addition, PA-13 was able to eradicate most bacteria within 2 h at MIC (and MBC). After 24 h treatment, regrowth was observed when bacteria were incubated with PA-13 at MIC, whereas PA-13 completely killed *P. aeruginosa* (no regrowth) at MBC within 4 h.

### Secondary structure of PA-13

**Figure 5:**
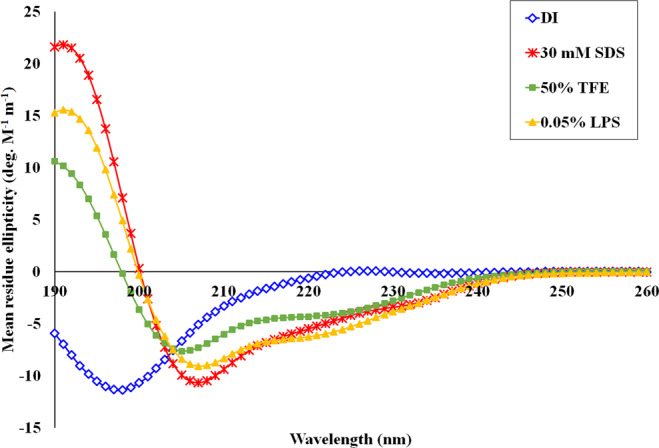
CD spectra of PA-13 in aqueous solution (blue), 30 mM SDS micelles (red), 50% TFE (green) and 0.05% LPS (orange). Circular dichroism spectra were measured at 25 °C using a Jasco-815 spectropolarimeter. The samples were loaded in a rectangular quartz cell (0.1 cm path length), and the spectra were recorded at a scanning speed of 10 nm/min in the wavelength range of 190 to 260 nm.

The secondary structure of PA-13 in different environments (aqueous solution, 30 mM SDS, 50% TFE and 0.05% LPS) was investigated by CD spectroscopy (Fig. 5). PA-13 formed random coil structures in aqueous solution as indicated by the presence of a strong minimum peak near 200 nm. In contrast, PA-13 in 30 mM SDS, 50% TFE, and 0.05% LPS displayed an increase in the mean residue ellipticity at 208 and 222 nm, consistent with the formation of α-helical structures. These results suggested that PA-13 formed an amphiphilic α-helical structure in membrane-mimetic environments.

### Influence of salts in the antibacterial activity

The antibacterial activity of PA-13 in the presence of different physiological salts was investigated. As shown in Table 2, the peptide retained stable antimicrobial activity against *P. aeruginosa* ATCC strain 27853 in the presence of NH^4^^+^, Zn^2+^, Fe^3+^ and Ca^2+^, while the MIC of PA-13 increased 2-fold in the presence of K^+^. These results suggested that cation valence [monovalent (NH^4^^+^, K^+^), divalent (Zn^2+^, Ca^2+^) and trivalent (Fe^3+^)] had little to no effect on the strength of PA-13’s antimicrobial activity. There was a marked decrease in the antimicrobial activity in the presence of Na^+^ and Mg^2+^. Therefore, some salts could compromise the MIC values of PA-13.

### Membrane permeability and depolarization

**Figure 6:**
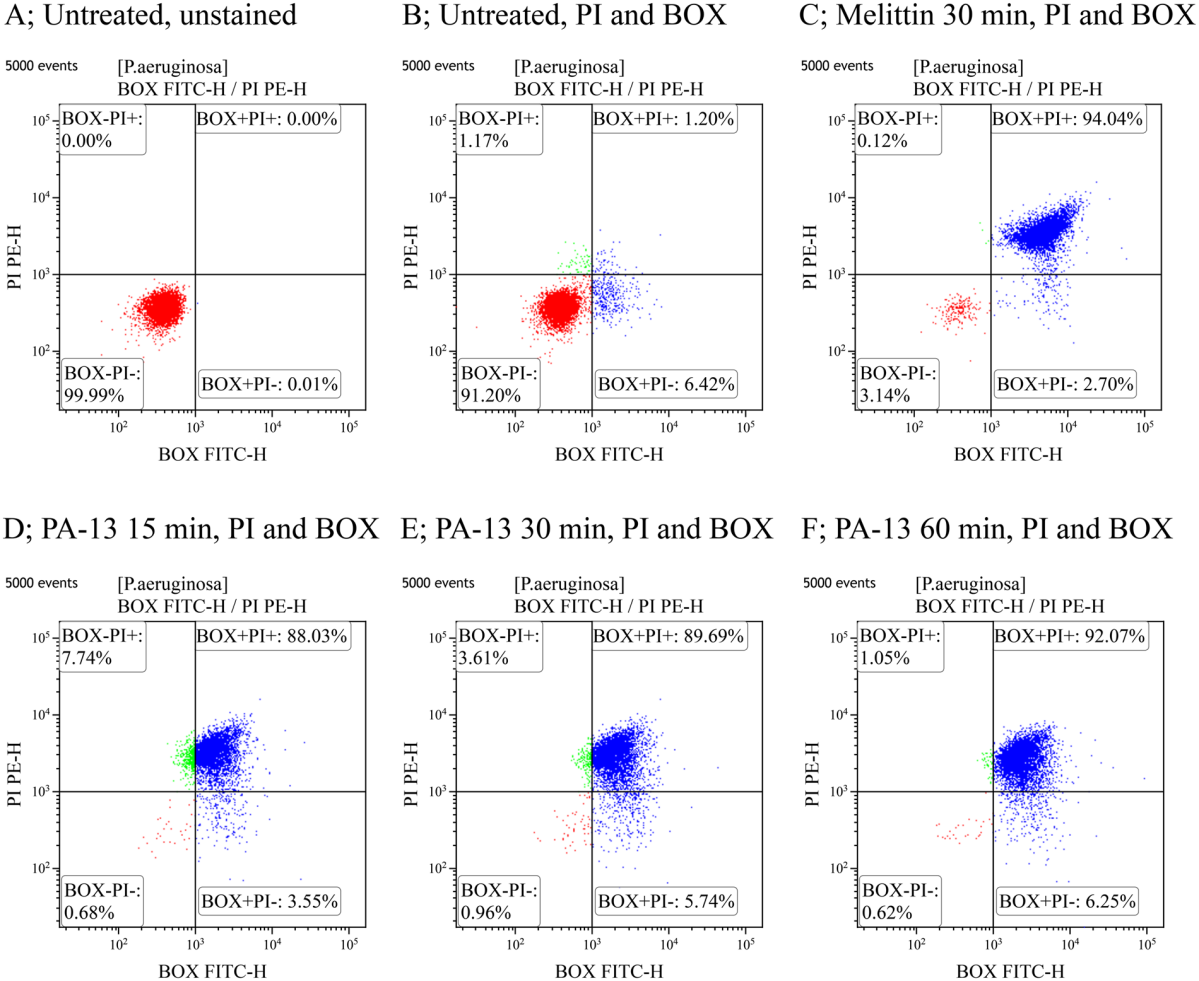
Flow cytometry analysis of *P. aeruginosa* treated with PA-13 or melittin (control). (**A**) Untreated *P. aeruginosa* without PI and BOX. (**B**) Untreated *P. aeruginosa* with PI and BOX stained. The effect of melittin at 1 × MIC for 30 min (**C**) and PA-13 at 1 × MIC for 15, 30 and 60 min (**D–F**) on membrane permeability (PI) and membrane potential (BOX) of *P. aeruginosa*. The percentage of cell populations that fell in each gate are shown in the four corners of each plot.

The mechanism of action at the membrane level of PA-13 was studied using flow cytometry. PI fluorescently stains the nucleic acids in cells following cytoplasmic membrane disruption, while BOX incorporates into depolarized cells where it binds to lipid-rich intracellular compounds and increases fluorescence. Unstained cells (Fig. 6A) are used to set the negative population. In the absence of PA-13, 91.20 ± 3.73% of *P. aeruginosa* bacteria showed no PI and the BOX fluorescent signal indicated that cell membranes were intact (Fig. 6B). Melittin was used as a positive control in the experiment as it was known to target the membranes of Gram-negative bacteria. As expected, the flow cytometric analysis showed that melittin induced high levels of permeabilization and depolarization at 1 × MIC for 30 min (94.04 ± 3.25) (Fig. 6C). PA-13 treatment induced dose- and time-dependent permeabilization and depolarization. At 1 × MIC, PA-13 quickly induced high levels of permeabilization and depolarization with 88.03 ± 3.02%, 89.69 ± 5.25%, and 92.07 ± 5.15% in 15, 30 and 60 min, respectively (Fig. 6D–F). At 0.5 × MIC, PA-13 still induced high levels of membrane permeability and depolarization (72.75 ± 1.08%, 75.30 ± 2.68% and 81.66 ± 3.35% in 15, 30 and 60 min, respectively) (Fig. S4). These results suggested that PA-13 potently damaged the *P. aeruginosa* membrane by permeabilization and depolarization in a dose- and time-dependent manner.

### Membrane-penetrating activity

**Figure 7:**
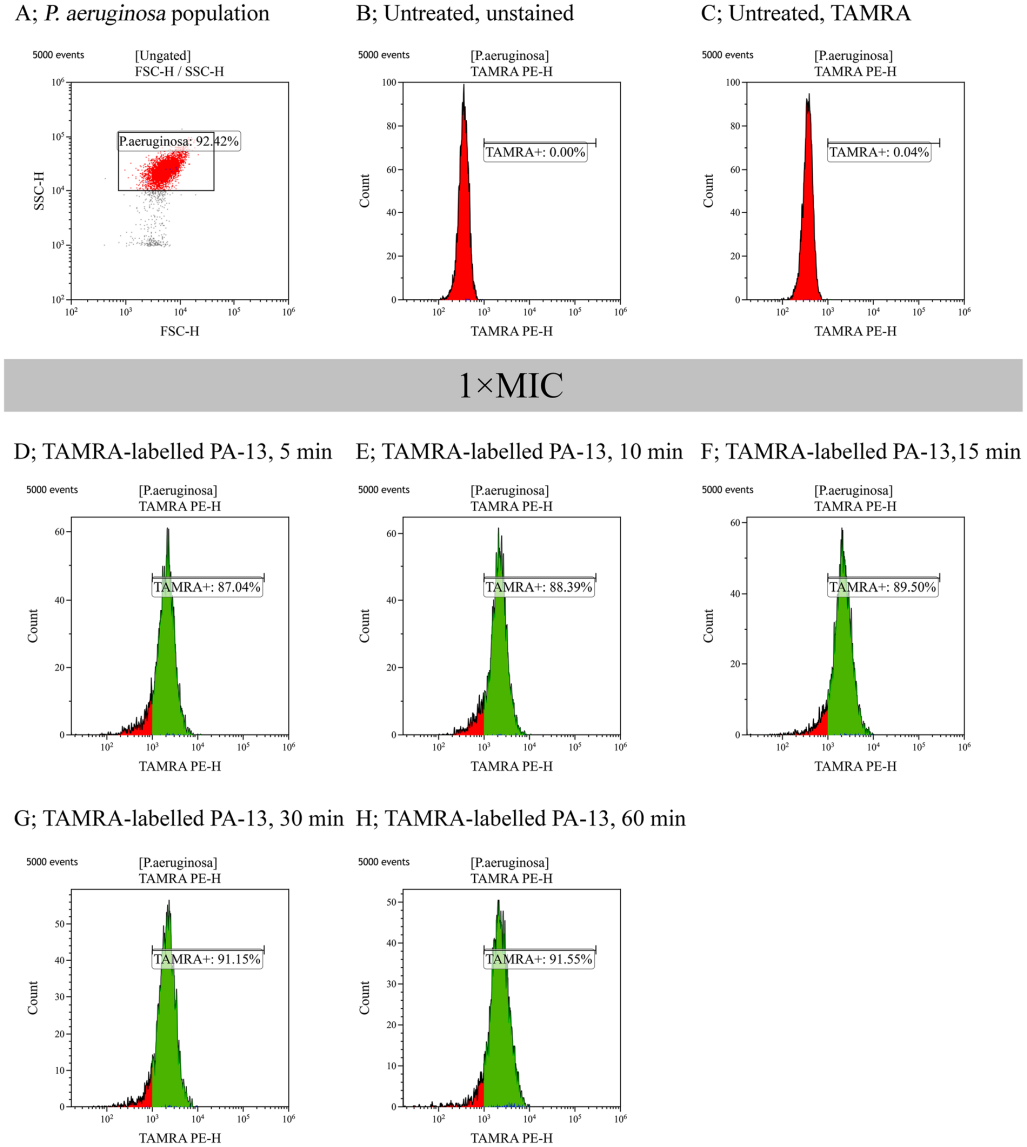
Binding of TAMRA-labelled PA-13 at 1 × MIC to *P. aeruginosa* ATCC 27853 as measured by flow cytometry. (**A**) *P. aeruginosa* cell population (**B**) Untreated *P. aeruginosa* without staining. (**C**) Untreated *P. aeruginosa* stained with TAMRA. Membrane-penetrating activity of TAMRA-labelled PA-13 after 5 min (**D**), 10 min (**E**), 15 min (**F**), 30 min (**G**) and 60 min (**H**) incubation. The percentage of cell populations are shown in the center of each plot.

**Figure 8:**
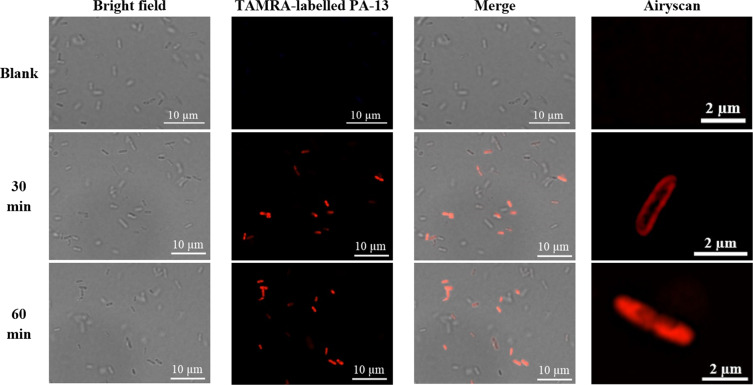
Localization of TAMRA-labelled PA-13 on *P. aeruginosa* ATCC 27853 at 30 and 60 min as observed by confocal microscopy.

The interaction of TAMRA-labelled PA-13 with the membrane of *P. aeruginosa* was investigated by flow cytometry and confocal microscopy. 92.42% of the *P. aeruginosa* cell population was analyzed in this study, as shown in Fig. 7A. In the absence of TAMRA-labelled PA-13, there was no fluorescent signal (Fig. 7B,C) indicating non-autofluorescence and healthy bacterial cells. The flow cytometry results indicated that TAMRA-labelled PA-13-treated *P. aeruginosa* cells exhibited red fluorescence with 87.04 ± 3.00%, 88.39 ± 2.64%, 89.5 ± 3.37%, 91.15 ± 4.44% and 91.55 ± 2.91% at 5, 10, 15, 30 and 60 min, respectively (Fig. 7D–H). These results suggested that PA-13 exhibited rapid binding and penetration activities against *P. aeruginosa* (within 5 min) in a time-dependent manner. Results from confocal microscopy also indicated that TAMRA-labelled PA-13 exhibited an ability to penetrate *P. aeruginosa* cell membranes within 5 min (data not shown). In addition, TAMRA-labelled PA-13 showed clear penetration through *P. aeruginosa* cell membranes and accumulation in bacterial cytoplasm after 30 and 60 min of treatment (Fig. 8).

### Scanning and transmission electron microscopy

**Figure 9:**
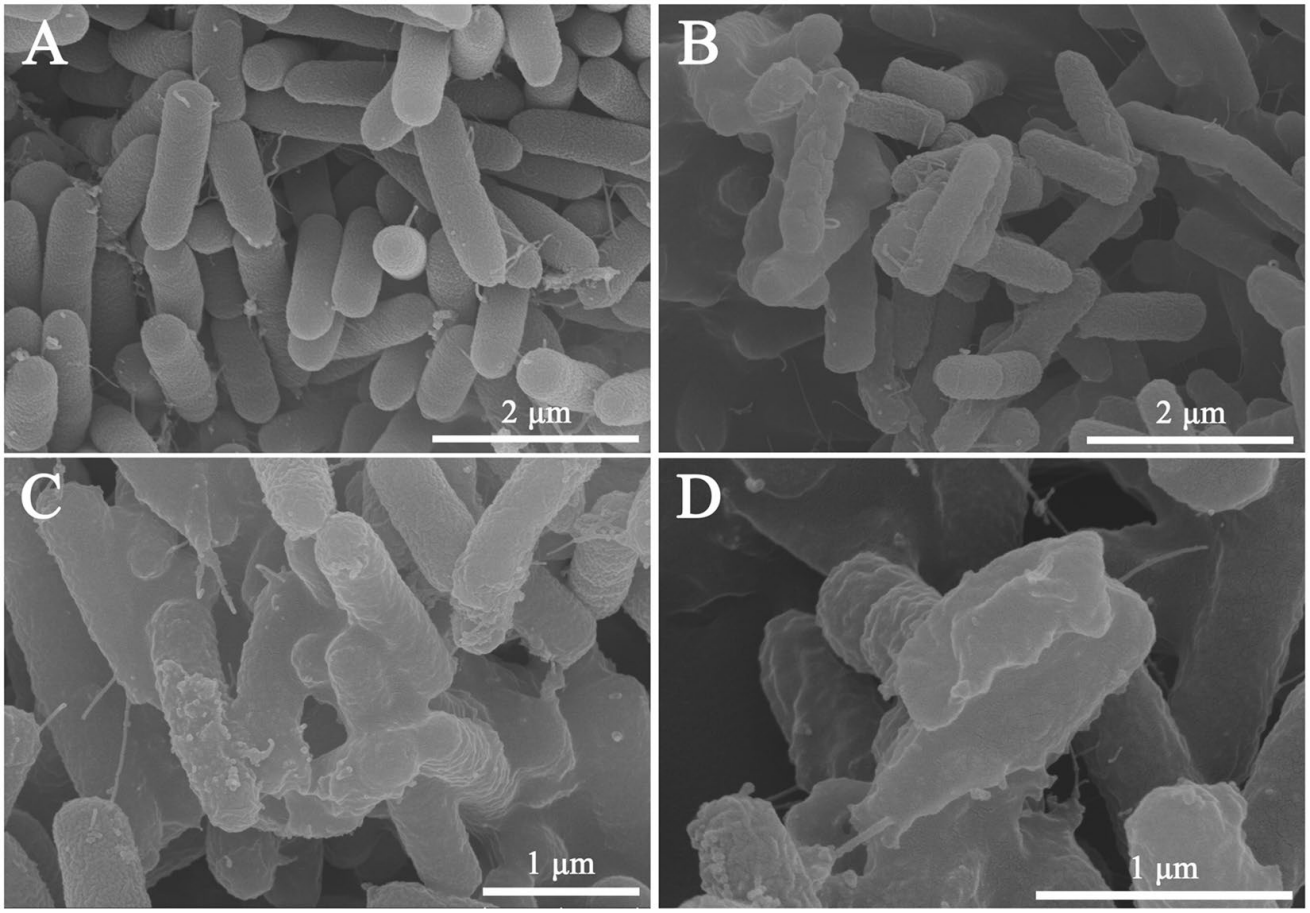
Scanning electron microscopic micrographs of *P. aeruginosa* ATCC 27853 treated with PA-13. (**A**) No peptide (Control); (**B**–**D**) Treated with 0.5 × MIC PA-13 for 2 h.

To observe cell morphologic changes after peptide treatments, SEM was conducted. As shown in Fig. 9A, bright and smooth surfaces were observed on the untreated *P. aeruginosa* cells (controls). Treatment with PA-13 for 2 h resulted in significant membrane roughening, corrugation, and damage of bacterial morphology (Fig. 9B–D).

**Figure 10:**
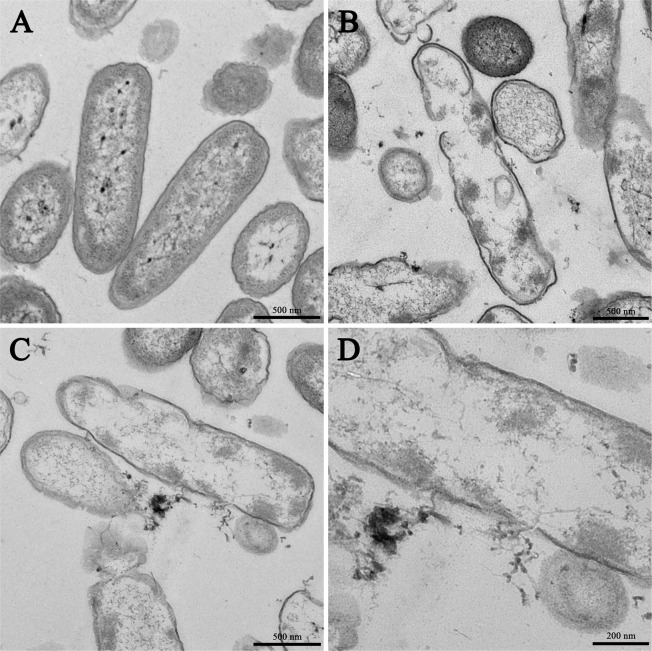
Transmission electron microscopic micrographs of *P. aeruginosa* ATCC 27853 treated with PA-13. (**A**) No peptide (Control); (**B**–**D**) Treated with 0.5 × MIC PA-13 for 2 h.

In addition to SEM, TEM was employed to study membrane integrity and intracellular alterations of *P. aeruginosa* cells both before and after treatment with PA-13. As shown in Fig. 10A, intact cell membranes and full intracellular contents were observed in untreated *P. aeruginosa* cells. In contrast, several structural alterations including obvious cytoplasmic clear zones, disrupted cell membrane with visible pores and leakage of cellular contents were observed after PA-13 treatment (Fig. 10B–D).

### LPS neutralization

**Figure 11:**
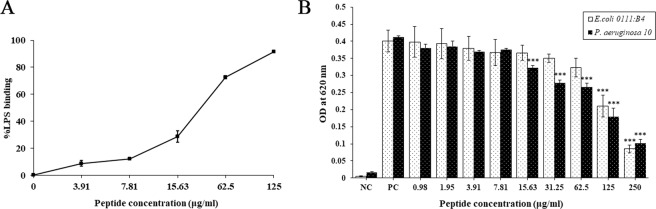
Anti-inflammatory activity of PA-13. (**A**) the concentration-response curves of LPS binding (*E. coli* O111:B4) by PA-13 as determined by LAL assay. (**B**) The dose-dependent inhibition of LPS-mediated TLR4 activation by PA-13, as determined by HEK-Blue™ hTLR4 cells. One-way ANOVA and Tukey’s Honestly Significant Difference (HSD) tests were employed. ***p-value <0.001.

After establishing the peptide’s potent antimicrobial activity, anti-inflammatory activity was investigated. LPS is a major component of the outer membrane in Gram-negative bacteria and constitutes an important barrier, while also triggering inflammatory effects. The ability of PA-13 to bind to the LPS of *E. coli* O111:B4 was determined using a LAL assay. PA-13 displayed dose-dependent LPS binding activity (Fig. 11A). In addition, PA-13 showed dose-dependent inhibition of LPS-mediated TLR4 activation as determined by HEK-Blue™ hTLR4 cells, especially toward the LPS of *P. aeruginosa* (Fig. 11B). From these results, we conclude that PA-13 was able to bind and neutralize LPS in a dose-dependent manner. Taken together, PA-13 appears to be a promising anti-inflammatory agent.

## Discussion

The emergence in pathogenic bacteria of resistance to conventional antibiotics has become a serious threat to public health. This crisis leads to an urgent need to develop novel classes of antimicrobial molecules^33^. Antimicrobial peptides (AMPs) are important components of the innate immune system as a first line of defense against microbial infections^34^. They are potential antimicrobial agents due to their multi-functional properties which result in broad-spectrum antimicrobial activity and low likelihood of inducing bacterial resistance^35^. Along with their many desirable properties, AMPs also may exhibit unfavorable properties such as cytotoxic and/or hemolytic effects, the loss of antimicrobial activity by salt, and high cost of production^17^. However, these barriers are being overcome via many approaches, notably the modification of peptide sequences. Therefore, the development of AMP-based antibiotics remains achievable though challenging.

Membrane-interacting AMPs with α-helical structures have gained much attention regarding AMP design and development. Cathelicidins, AMPs of the innate immune system of many vertebrates, have a broad spectrum of antimicrobial activity against bacteria, enveloped viruses and fungi^11,34,36^. Aureins, a family of AMPs found in skin secretions of several species of Australian tree frogs of the genus *Litoria*, exhibit antibacterial, antifungal, antiviral and anticancer activity^15,37^. Cathelicidin and aurein are classified as a promising family of α-helical AMPs. Their structure-activity relationships, simple α-helical architectures and broad-spectrum activities make them very attractive for use in AMP design and development.

In this study, the sequence and structural information of α-helical cathelicidin and aurein were used to design P0 and A0 parent peptides, respectively. These parent peptides, with imperfect amphipathic structures, showed no antimicrobial activity against all tested bacteria. Many reports note that truncation and amino acid substitution is an effective method for developing candidate AMPs^8,27,38^. We therefore modified parent peptide sequences by truncation of unstructured regions and amino acid substitution. Their derivatives, especially P7, showed perfect amphipathicity and higher hydrophobicity than the parental peptides with significantly improved antimicrobial activity. These results suggested that the unstructured region at positions 1 to 3 did not affect antimicrobial activity, but that both amphipathicity and hydrophobicity played crucial roles in antimicrobial activity. In addition, the potent antimicrobial activity of P7 may have resulted from the bulky side chain of tryptophan at position 7. Since this located on the hydrophobic/hydrophilic interface, the interaction of peptides with bacterial membranes is increased^8^.

Combinations of two or more native peptides, or hybrid peptide derivatives, have received great attention, as this can enhance their antimicrobial properties^22^. Since P7 showed much higher antimicrobial activity than A3, the sequence of P7 was maintained with its activity further improved by hybridizing with A3 at the C-terminus. P7A3 displayed higher antimicrobial activity against all tested bacteria when compared with P7 and A3 alone, however P7A3 also displayed the strongest hemolytic activity. Our results indicated that the high positive charge and hydrophobicity of P7A3 correlated with both its antimicrobial and hemolytic activities. To minimize toxicity, truncated derivatives were designed to identify the shortest amino acid sequence which retained the potent antimicrobial activity while reducing toxicity. It has been shown that the N-terminal α-helical domain of peptides such as PMAP-36 is its active region and has the ability to interact with and penetrate the bacterial membranes^8^. Moreover, an unstructured region (position 18 to 23) was observed in the C-terminus of the ribbon structure of P7A3. Therefore, the C-terminus of the hybrid peptide was truncated. The truncated derivatives of the hybrid analogue (PA-14 and -13) displayed high antimicrobial activity against both Gram-negative and -positive bacterial strains with relatively decreased toxicity. Our studies indicated that increasing amphipathicity (with suitable positive charges) and hydrophobicity improved antimicrobial activity and selectivity. Among truncated derivatives, the shortest peptide (PA-13) showed potent antimicrobial activity along with low toxicity against both hRBCs and L929 cells. Differences in amino acid sequence and position have been suggested to be important for antimicrobial activity. Importantly, the bulky side chain of tryptophan at the hydrophobic/hydrophilic interface of all truncated derivatives played an important role in facilitating the interaction of peptides with bacterial membranes. The antimicrobial activity and toxicity of AMPs are related to multiple physio-chemical properties, for instance the length, charge, hydrophobicity, amphipathicity and hydrophobic/hydrophilic angle. Thus, adjusting one can cause alterations to the others and so it is difficult to highlight the influence of a single factor for the activity^4^.

The therapeutic index is a widely employed parameter to indicate the specificity of AMPs. It is calculated as the ratio of MHC (hemolytic activity) and MIC (antimicrobial activity)^27^. Thus, larger TI values indicate greater antimicrobial specificity. Among all derivative peptides, P7 exhibited the highest TIs (8.78 and 2.06 against Gram-negative and -positive bacteria, respectively). The hybrid peptide P7A3 displayed high antimicrobial activity coupled with strong hemolytic activity. Hence its TI was low (0.09 and 0.31 for Gram-negative and -positive bacteria, respectively). PA-18, a truncated form containing the entire α-helical structure of the hybrid peptide, displayed comparable specificity with P7A3. Among truncated derivatives, PA-13 exhibited the highest TI against all tested bacterial strains suggesting that it had increased selectivity toward bacterial cells compared with hRBCs, and implying it had a wider therapeutic window. Our studies demonstrated that an increase of TI mainly resulted from a reduction of hemolytic activity, and indicated that truncation at the C-terminus of peptides affected their hemolytic activity. Therefore, the potent antimicrobial activity with low host toxicity of PA-13 reflects an attractive direction for AMP development.

It has been suggested that the propensity of AMPs to form an amphipathic α-helix in membrane-mimetic environments is the key to their membrane-disruptive activity^27^. In the current study, the secondary structure of PA-13 in water, membrane-mimicking environments and LPS was analyzed using CD spectra. The results indicated that PA-13 exhibited a random coil in aqueous environments. In 30 mM SDS, 50% TFE and 0.05% LPS of *P. aeruginosa*, PA-13 exhibited a typical α-helical structure, with more helical content in 30 mM SDS than in 50% TFE and 0.05% LPS. This result was consistent with previous studies indicating that the structural change of peptides from a random coil to an α-helix structure might be one of the most important factors determining peptide function at bacterial cell membranes, including antimicrobial activity^39^.

Previous studies have demonstrated that a positive charge facilitates antimicrobial peptide binding to the negatively-charged bacterial membranes, and/or LPS, via electrostatic interactions^40^. However, other positively charged molecules, including salts, can weaken this electrostatic interaction. The antibacterial activity of PA-13 in the presence of different physiological salts was investigated. PA-13 retained its antimicrobial activity in the presence of NH^4+^, Zn^2+^, Fe^3+^, Ca^2+^ and K^+^, suggesting that cation valence [monovalent (NH^+^, K^+^), divalent (Zn^2+^, Ca^2+^) and trivalent (Fe^3+^)] had little or no effect on the strength of PA-13’s antimicrobial activity. This might be due to the bulky side chain of tryptophan, which could enhance the affinity of this antimicrobial peptide for the bacterial membrane and contribute to its strong antibacterial activity in the presence of salts^8^. However, there was a marked decrease in the antibacterial activity of PA-13 against *P. aeruginosa* in the presence of Na^+^ and Mg^2+^. Mg^2+^ compromised the antimicrobial activity, possibly due to competition with cationic peptide for binding with LPS molecules on bacterial outer membranes. The presence of Na^+^ could hinder electrostatic interactions and decrease the binding efficacy of the peptide. This might be attributed to the first step in antimicrobial activity, ultimately compromising the killing efficiency of a peptide^41,42^. However, this is in consistent with the results of other studies^43,44^. While F4 peptide retains killing activity in the presence of salts at physiologic concentrations, except Na^+^ and Mg^2+^, it still displays effective antimicrobial potency in the mouse model^45^.

Although the exact mechanism of action of AMPs has not been established, it has been proposed that the cytoplasmic membranes of bacteria are the main target of these peptides via their disruption or the formation of pore/ion channels^35^. To assess membrane-penetrating activity and localization, TAMRA-labelled PA-13 was incubated with *P. aeruginosa* and the bacteria investigated by flow cytometry and confocal microscopy. The results indicated that PA-13 exhibited time-dependent penetrating activity. PA-13 rapidly punctured the bacterial membranes (within 5 min at 1 × MIC). The results were strongly in accordance with localization studies. That is, TAMRA-labelled PA-13 clearly localized on *P. aeruginosa* membranes and then accumulated in bacterial cytoplasm. After inserting into the cytoplasmic membrane, PA-13 potentially disrupted the membrane integrity in dose- and time-dependent manner. This appeared concordant with the collapse of membrane electrical potential as observed by flow cytometry. The results of SEM and TEM further confirmed the killing mechanisms of PA-13 being membrane rupture and pore formation, leading to the leakage of intracellular contents and cell death. The fluorophore-PEN conjugates displayed altered modes of membrane interaction with increased insertion into the core of the model cell membrane and exert membrane-thinning effects without effecting membrane-penetrating ability^46^. This agrees with our results, showing the penetrating ability of TAMRA-labelled PA-13 in a time- dependent manner and that the unlabeled PA-13 behaved similarly to the labeled compound determined by circular dichroism and flow cytometry (data not shown).

Lipopolysaccharide from bacterial endotoxin, a major component of the outer membrane of all Gram-negative bacteria, can cause inflammation, sepsis and shock^47^. Previous research demonstrated that the peptide-LPS interaction strongly promotes bacterial cell death and reduces inflammation. Anti-inflammatory activity of AMPs can occur as a result of neutralization of biological inflammatory cytokines or inhibition of their production^48^. Our studies revealed that PA-13 was able to bind and neutralize LPS, the negatively charged molecules constituting an important barrier in the membranes of Gram-negative bacteria, in a dose-dependent manner. Furthermore, PA-13 showed dose-dependent inhibition of LPS-mediated, Toll-like receptor activation, which inhibited the release of pro-inflammatory cytokines and down-regulated further severe inflammation. Therefore, PA-13 appears to be a very promising anti-inflammatory agent.

There are several reports on the relationship between *in vitro* activity and *in vivo* efficacy and safety of antimicrobial peptides^49–54^. A novel peptide, BSN-37, exhibits strong antibacterial activity against *S*. Typhimurium and significantly inhibits the proliferation of intracellular *S*. Typhimurium with no associated toxicity to the eukaryotic cells^49^. A broad-spectrum antimicrobial peptide, GL13K, with low cytotoxicity *in vitro* exhibits a low level of hemolysis and anti-inflammatory activity *in vivo* in a mouse model of inactivated LPS-induced sepsis^50,51^. Although less peptide binding is observed, GL13K is still active in physiological salts conditions^52^. The *in vivo* protective effect of buforin II in an experimental rat model of *A. baumannii* sepsis confirms the *in vitro* effectiveness of this peptide^53^. The proline-rich, bovine innate immune peptide, Bac5, is able to kill some mycobacterial species and supports macrophage activation *in vitro* in synergy with *Mycobacterium marinum*. Peptide Bac5 is able to slow *M. marinum* infections of zebrafish^54^. The *in vitro* characterization of antimicrobial peptides may be applicable as predictors of *in vivo* efficacy and safety. Our *in vitro* data may, similarly, predict the *in vivo* efficacy and safety of PA-13. To continue the clinical development of this peptide, studies of efficacy and safety in animal models will be conducted.

In conclusion, among rationally designed, short α-helical hybrid peptides inspired by cathelicidin and aurein, PA-13 exhibited potent antibacterial activity against *P. aeruginosa*, including multidrug resistant strains, via penetration through the bacteria’s membrane and causing depolarization, permeabilization and rupture of membranes, and subsequent leakage of intracellular contents and cell death. The peptide displayed low hemolytic and cytotoxic activities against mammalian cells suggesting a therapeutic potential. Furthermore, PA-13 showed anti-inflammatory activity via LPS neutralization. Our research supports the usefulness of rational design of hybrid peptides as promising candidates for development of novel antimicrobial agents.

## Supplementary information

Supplementary information.
