# Supplementary material for: A novel, rationally designed, hybrid antimicrobial peptide, inspired by cathelicidin and aurein, exhibits membrane-active mechanisms against Pseudomonas aeruginosa
Source: Sci Rep. 2020 Jun 4;10:9117. doi: 10.1038/s41598-020-65688-5 (PMC7272617; doi:10.1038/s41598-020-65688-5)
Supplement: Supplementary file 1 — Supplementary information. [file 41598_2020_65688_MOESM1_ESM.pdf]

Supplementary information

A novel, rationally designed, hybrid antimicrobial peptide, inspired by cathelicidin and aurein, exhibits membrane-active mechanisms against *Pseudomonas aeruginosa*

Natthaporn Klubthawee[1], Poom Adisakwattana[2], Warunee Hanpithakpong[3], Sangdao Somsri[1] and Ratchaneewan Aunpad[1,*]

[1]Graduate Program in Biomedical Sciences, Faculty of Allied Health Sciences, Thammasat University, Pathum Thani, Thailand

[2]Department of Helminthology, Faculty of Tropical Medicine, Mahidol University, Bangkok, Thailand

[3]Department of Clinical Pharmacology, Mahidol Oxford Tropical Medicine Research Unit, Faculty of Tropical Medicine, Mahidol University, Bangkok, Thailand

*Corresponding author

Email: aratchan@tu.ac.th; +66829869213

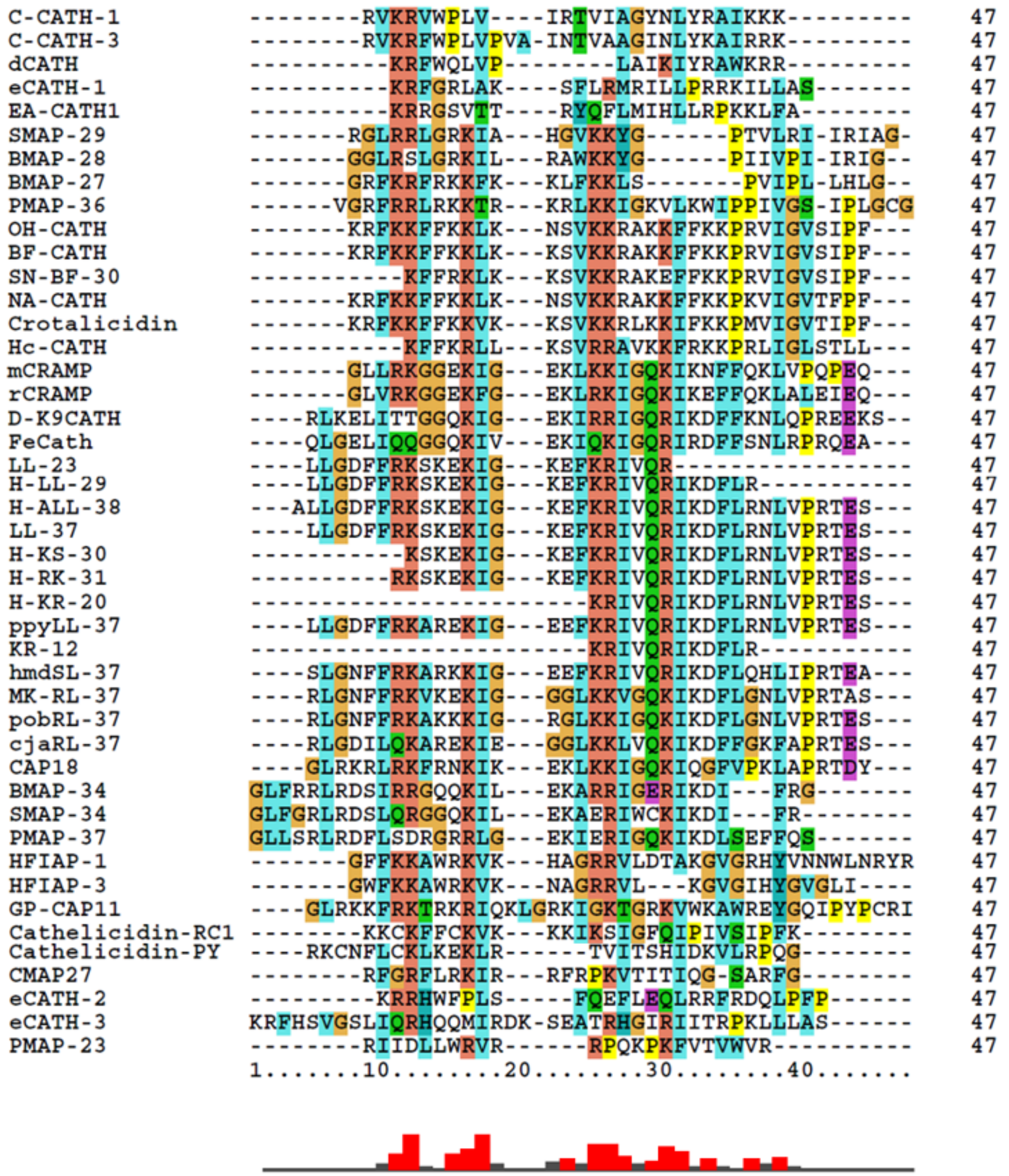

Figure S1 Multiple sequence alignments of 45 α-helical cathelicidins identified in the Antimicrobial Peptide Database (APD) using ClustalX2 program. Each residue in the alignment is assigned a color if the amino acid profile of the alignment at that position meets the minimum criteria specific for the residue type (http://www.jalview.org/help/html/colourSchemes/clustal.html).

By default, hydrophobic residues are shadowed in blue, positively charged in red, negatively charged in magenta and polar in green. Special residues such as glycine, proline and aromatic residues are colored in orange, yellow and cyan, respectively. The conserved sequences were used to build a scaffold to design a P0 parent peptide shown in red bar graphs.

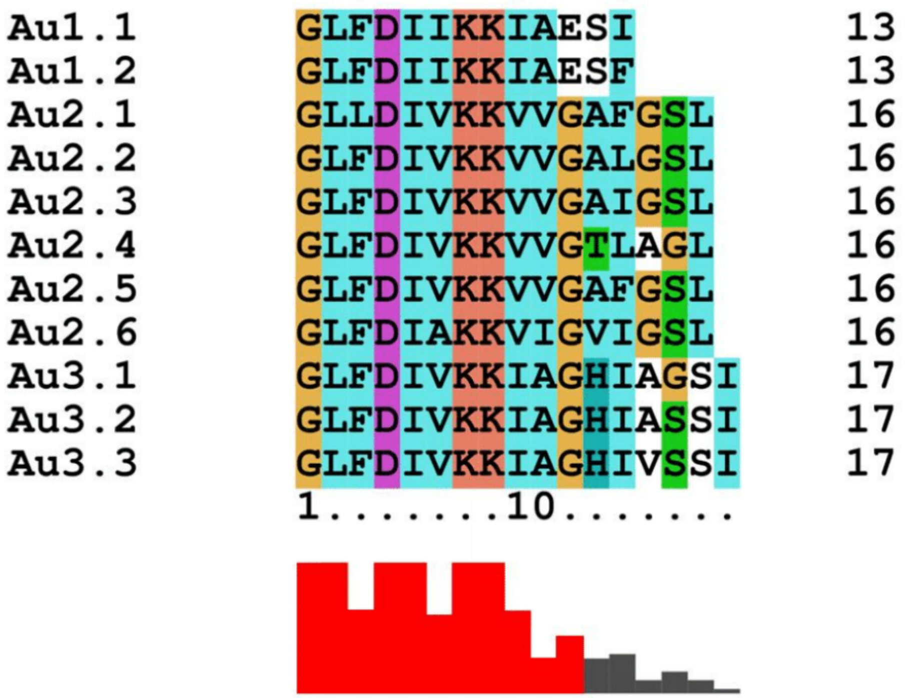

Figure S2 Multiple sequence alignment of 11 α-helical aureins identified in the Antimicrobial Peptide Database (APD) using ClustalX2 program. Each residue in the alignment is assigned a color if the amino acid profile of the alignment at that position meets the minimum criteria specific for the residue type (http://www.jalview.org/help/html/colourSchemes/clustal.html).

By default, hydrophobic residues are shadowed in blue, positively charged in red, negatively charged in magenta and polar in green. Special residues such as glycine, proline and aromatic residues are colored in orange, yellow and cyan, respectively. The conserved sequences were used to build a scaffold to design a P0 parent peptide shown in red bar graphs.

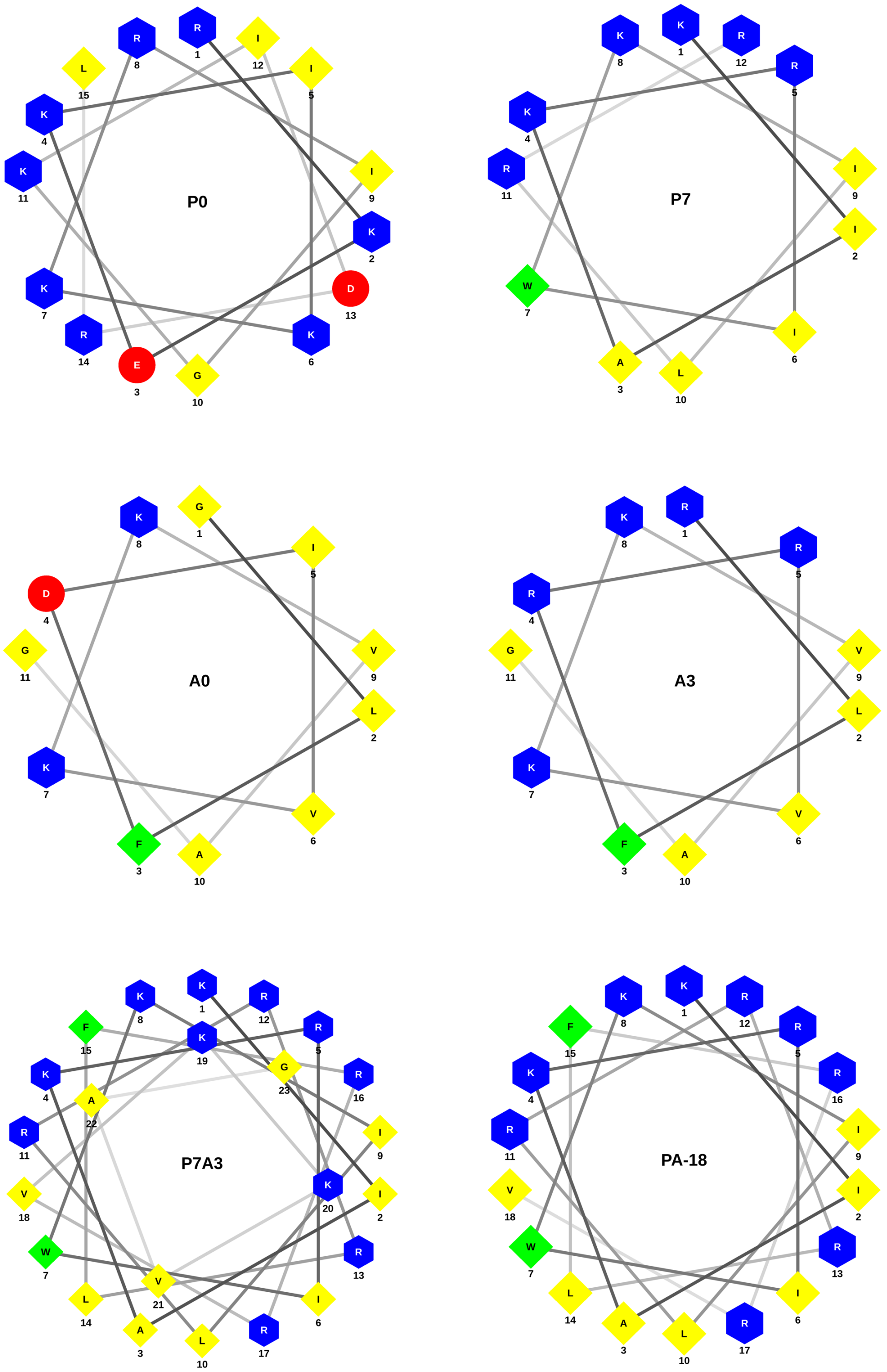

Figure S3. Helical wheel projections of P0, P7, A0, A3, P7A3 and PA-18.

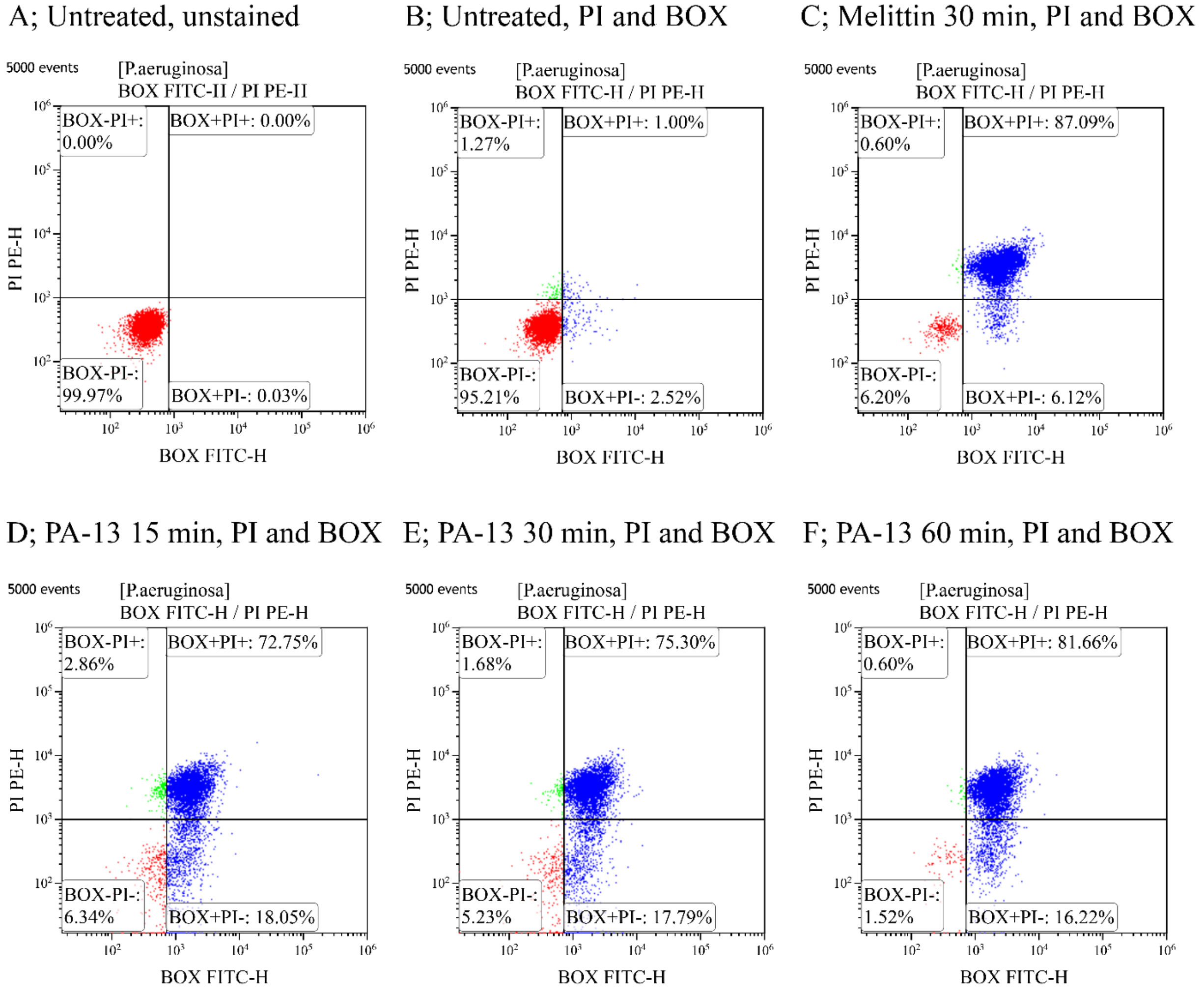

Fig S4. Flow cytometry analysis of *P. aeruginosa* treated with PA-13 or melittin (control). (A) Untreated *P. aeruginosa* without staining with PI and BOX. (B) Untreated *P. aeruginosa* staining with PI and BOX. The effect of melittin at 0.5×MIC for 30 min (C) and PA-13 at 0.5×MIC for 15, 30 and 60 min (D-F) on membrane permeability (PI) and membrane potential (BOX) of *P. aeruginosa*. The percentage of cell populations that fell in each gate is shown in the four corners of each plot.

Table S1 Rational design of novel AMPs by using a template-modified strategy. Positional frequency of amino acid residues derived from conserved sequences of 45 α-helical cathelicidins.

	**Position of novel AMPs (N- to C-terminus)**														
	**1**	**2**	**3**	**4**	**5**	**6**	**7**	**8**	**9**	**10**	**11**	**12**	**13**	**14**	**15**
	R^{16}	K^{24}	E^{11}	K^{32}	I^{20}	K^{10}	K^{25}	R^{19}	I^{21}	Q^{19}	K^{16}	I^{26}	D^{17}	R^{16}	L^{18}
	K^{12}	R^{10}	K^{10}	R^{4}	V^{7}	E^{9}	R^{7}	K^{18}	R^{5}	K^{7}	R^{14}	L^{6}	K^{9}	K^{6}	I^{7}
			R^{7}	L^{4}	L^{6}	S^{7}				I^{4}		F^{6}	R^{5}		F^{7}
			Q^{5}			G^{4}									
			P^{2}			A^{3}									
Novel sequence (P0)	R	K	E	K	I	K	K	R	I	Q	K	I	D	R	L

Table S2 Rational design of novel AMPs by using a template-modified strategy. Positional frequency of amino acid residues derived from conserved sequences of 11 aureins.

	Position of novel AMPs (N- to C-terminus)										
	**1**	**2**	**3**	**4**	**5**	**6**	**7**	**8**	**9**	**10**	**11**
	G^{11}	L^{11}	F^{10}	D^{11}	I^{11}	V^{8}	K^{11}	K^{11}	V^{6}	A^{5}	G^{9}
			L^{1}			I^{2}			I^{5}	V^{5}	
						A^{1}				I^{1}	
Novel sequence (A0)	G	L	F	D	I	V	K	K	V	A	G

Table S3 Amino acid sequence of peptides and their key physicochemical parameters.

Peptide	Sequence	Theoretical MW	Measured MW[a]	aa[b]	Net charge	uH[c]	Pho%[d]
P0	RKEKIKKRIGKIDRL-NH_2	1881.326	1880.37	15	+6	0.443	26%
P7	KIAKRIWKILRR-NH_2	1581.021	1580.06	12	+6	0.801	50%
A0	GLFDIVKKVAG-NH_2	1146.390	1145.42	11	+1	0.595	54%
A3	RLFRRVKKVAG-NH_2	1329.653	1328.69	11	+5	0.661	45%
P7A3	KIAKRIWKILRRRLFRRVKKVAG-NH_2	2892.659	2891.71	23	+11	0.451	47%
PA-18	KIAKRIWKILRRRLFRRV-NH_2	2409.055	2408.10	18	+9	0.454	50%
PA-17	KIAKRIWKILRRRLFRR-NH_2	2309.922	2308.97	17	+9	0.459	47%
PA-16	KIAKRIWKILRRRLFR-NH_2	2153.734	2152.78	16	+8	0.546	50%
PA-15	KIAKRIWKILRRRLF-NH_2	1997.546	1996.59	15	+7	0.551	53%
PA-14	KIAKRIWKILRRRL-NH_2	1850.369	1849.41	14	+7	0.699	50%
PA-13	KIAKRIWKILRRR-NH_2	1737.209	1736.25	13	+7	0.678	46%

[a] MW, molecular weight (g/mol) measured by mass spectroscopy (MS).

[b] aa, number of amino acids.

[c] µH, the mean hydrophobic moment obtained from the website: http://heliquest.ipmc.cnrs.fr/.

[d] Pho%, the percentage of hydrophobic residues.
